# Evaluation of Root Canal Configuration of Mandibular First Molars in a Palestinian Population by Using Cone-Beam Computed Tomography: An Ex Vivo Study

**DOI:** 10.1155/2014/583621

**Published:** 2014-08-13

**Authors:** Raed Hakam Mukhaimer

**Affiliations:** Department of Conservative Dentistry & Endodontics, Dental School, Arab American University, P.O. Box 240, Jenin, Palestine

## Abstract

*Aim*. The purpose of this study was to investigate the number of canals and variations in root canal configuration in the mandibular permanent first molar teeth of a Palestinian population using cone-beam computed tomography (CBCT). *Methods*. A sample of 320 extracted double-rooted mandibular permanent first molars from Palestinian population was collected for this study and scanned with CBCT scanner. The following observations were made: number of root canals per root and canal configuration in each root based on Vertucci's classification. *Results*. Of the 320 mandibular first molars analyzed, 174 (54.4%) had three canals, 132 teeth (41.3%) had four canals, and only four teeth had two canals. The most common canal configuration in the mesial roots was Vertucci type IV (53.8%) followed by type II (38.8%). In the distal roots, the most prevalent canal configuration was Vertucci type I (57.5%) followed by type II ( 22.5%) and type III (10.6%). *Conclusion*. Our results showed that the number of canals and canal configuration in Palestinian population were consistent with previously reported data. The present study also indicates that CBCT is helpful as a diagnostic tool for the investigation of root canal morphology.

## 1. Introduction 

Variations in root canal morphology, especially in multirooted teeth, are a constant challenge for diagnosis and successful endodontic therapy. Root canal treatment can be highly guaranteed when all root canals are identified, thoroughly cleaned and shaped, and obturated with an inert filling material [1]. The clinician should be able to mentally visualize the pulp spaces from the coronal aspect to the apical foramen and should always be aware of the common internal root morphology and the possible variations which might be encountered. Otherwise, these anatomical variations may complicate endodontic treatment and compromise therapy outcomes [2, 3].

Mandibular first molars are the first permanent posterior teeth to erupt and are most prone to suffer from caries, leading to requirement of endodontic treatment [4]. It is generally accepted that mandibular first molars have two roots, a mesial root and a distal root and three or four canals, two in the mesial root and one or two in the distal root [5]. Variations in root and canal anatomy of mandibular first molar are not uncommon. The most relevant variable related to the number of roots is the presence of a third distolingual root. This macrostructure, first mentioned by Carabelli, was called radix entomolaris, which in general is smaller than mesial and distobuccal roots and can be separate from or partially fused with these other roots [6]. When located at the mesiobuccal side, this additional root is called the radix paramolaris [7].

Anatomical variations in the number and location of canals have been reported by several studies. These include the presence of a third canal in the mesial root [8], three canals in the distal root [9], and mandibular first molar with five, six, or even seven canals [10, 11].

Various techniques have been adopted to evaluate root canal morphology, and it has been reported that tooth demineralization and canal staining of extracted teeth are a very accurate method to detect root morphology [12]. Other techniques include access cavity preparation and radiographs with files placed in root canals [13], in vivo root canal treatment with radiographic evaluation [14], and macroscopic sectioning [15].

However, these methods had serious limitations as the relationship between the external structure and the pulp might get lost during preparation of study samples.

Advancements in the field of radiology have drawn attention to the use of computed tomography (CT) for imaging teeth. Technological advances have witnessed the invention of cone beam computerized tomography (CBCT) which was introduced in the field of endodontics in 1990 by Tachibana and Matsumoto [16]. CBCT scans were recently shown to be a valuable tool in various stages of endodontic treatment as they deliver immediate and accurate three-dimensional radiographic images. Preoperatively, these images offer superior diagnostic performance over conventional radiographic images and are used to improve the assessment of root canal systems as they provide information about the internal and external tooth anatomy including number and location of roots and canals, root and canal curvatures, size of the pulp chamber, and the degree of calcification [17]. Matherne et al. [18] investigated the use of CBCT scanning in identifying root canal systems and compared it with images obtained by using digital radiography. They concluded that CBCT images always resulted in the identification of greater number of root canal systems than digital images.

Additionally, CBCT technology aids in the diagnosis of endodontic and nonendodontic pathosis, assessing vertical root fractures, analysis of root resorption defects, and presurgical assessment of apicoectomy procedures [19, 20]. Postoperatively, CBCT scans can help in assessment of the technical quality of the root canal filling as well as healing of periapical lesions [21].

Many studies of root canal morphology in mandibular molars have been conducted because these teeth present a complex morphology that often complicates endodontic treatment. A review of the literature showed a lack of studies on root canal morphology of mandibular permanent first molar teeth in a Palestinian population. The purpose of this study was to investigate canal number and internal morphologies of permanent mandibular first molar using a sample of CBCT images obtained from extracted teeth of a Palestinian population.

## 2. Materials and Methods 

A sample of 320 extracted double-rooted mandibular permanent first molars from Palestinian population was collected from the tooth bank at the Department of Oral Surgery. Teeth included in this study were selected according to the following criteria: permanent mandibular first molars [22] with intact roots and fully formed apices. Teeth with broken roots, serious defects, and root canal fillings, posts, and crown restorations and teeth with signs of root resorption were excluded from the study. Reason or timing of extraction was not known. An ethical approval was obtained from the ethics committee of the university, and patients that were undergoing extraction signed an informed consent in regard to providing his/her tooth after being extracted for use in this study. Teeth were stored in 10% formalin for disinfection and were cleaned of calculus and remaining soft tissue by using an ultrasonic scaler. Then they were rinsed under running water and left to dry. As this study was carried out on extracted teeth, gender and age of the patients were not recorded. To integrate the samples positions, the teeth were fixed in some molds made of silicone impression molding materials (Zetaplus, Zhermack, Rovigo, Italy) in 6-tooth groups.

The mounted teeth were scanned with a CBCT scanner (MCT-1 [EX-2 F], Morita Manufacturing Corp, Kyoto, Japan) with image capture parameters set at 80 kV and 5.0 mA and an exposure time of 17.5 s. Tomographic sections of 1 mm in 3 planes (axial, coronal, and sagittal) were created. The CBCT images were analyzed using XoranCat software version 3.1.62 (Xoran Technologies, Ann Arbor, USA) on a Dell Precision T5400 workstation (Dell, Round Rock, TX), with a 32-inch Dell LCD in a darkroom. The contrast and brightness of the images were adjusted using the image processing tool in the software to ensure optimal visualization. All CBCT exposures were performed by a licensed expert radiologist.

**Figure 1:**
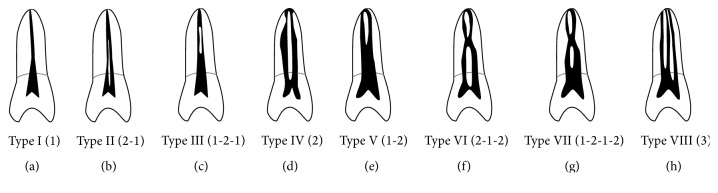
Classification of canal configurations according to Vertucci.

All mandibular first molars were thoroughly examined in the three planes (axial, sagittal, and coronal) at 1.0 mm intervals by continuously moving the toolbar from the floor of the pulp chamber to the apex. The following observations were registered: number of root canals per root and canal configuration in each root based on Vertucci's classification [23] (Figure 1). Two endodontists evaluated the radiographic images simultaneously to reach a consensus. In cases where a consensus was not reached, a third professional oral radiologist with endodontic experience was asked to perform a decisive evaluation.

## 3. Results 

**Figure 2:**
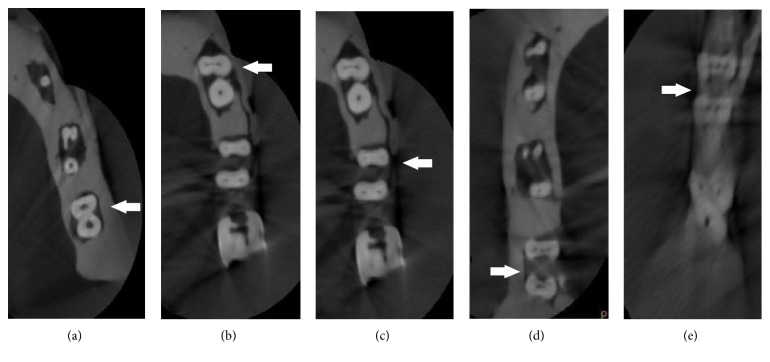
Cases of mandibular first molars with canal numbers in axial section; the white arrows indicate the examined tooth: (a) two-canal molar (one in mesial root and one in distal root); (b) three-canal molar (two in mesial root and one in distal root); (c) four-canal molar (two in mesial root and two in distal root); (d) four-canal molar (three in mesial root and one in distal root); (e) five-canal molar (three in mesial root and two in distal root).

The number and type of root canals of mandibular first molars are summarized in Table 1. Three hundred and twenty extracted mandibular first molars were analyzed. All selected teeth had two separate roots. In total, 174 (54.4%) of the mandibular first molars had three canals (mesiobuccal, mesiolingual, and distal), 132 teeth (41.3%) had four canals (mesiobuccal, mesiolingual, distobuccal, and distolingual), and only four teeth had two canals (1.2%). Analysis of the mesial root revealed one canal in 1.3%, two canals in 95.6%, and three canals in 3.1%. In the distal root, one canal was found in 57.5% and two canals were found in 42.5% of the examined roots (Table 2). Examples of canal distribution of examined teeth taken in an axial section are shown in Figure 2.

**Figure 3:**
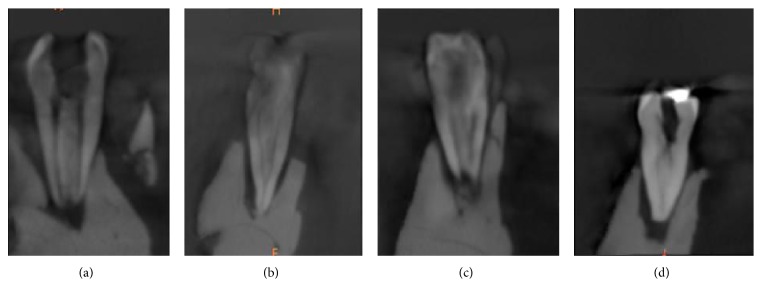
The top 4 canal configurations in the mesial roots of the examined teeth: (a) type IV, (b) type II, (c) type 3-2, and (d) type III.

**Figure 4:**
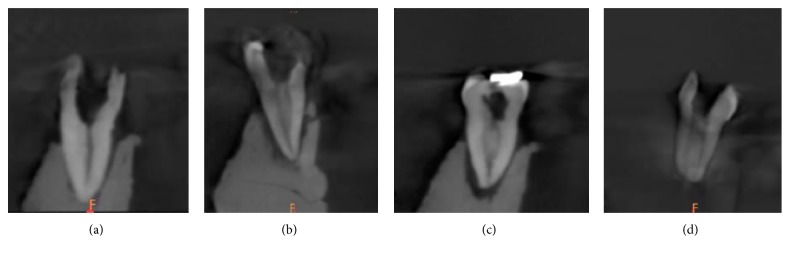
Cases of mandibular first molar with canal configuration in coronal section in the distal root: (a) type I, (b) type II, (c) type III, and (d) type IV.

Types of canal configuration for each root are summarized in Table 3. Of the examined teeth, the most common canal configuration in the mesial roots was Vertucci type IV (53.8%) followed by type II (38.8%) (Figure 3). In the distal roots, the most prevalent canal configuration was Vertucci type I (57.5%) followed by type II (22.5%) and type III (10.6%) (Figure 4).

## 4. Discussion 

Morphologic variation in the anatomy of the root canal system should always be considered at the beginning of root canal treatment. Each case, independent of which tooth is to be treated, should be examined clinically and radiographically in a thorough manner to detect possible anatomic variations.

It is generally accepted that a major cause of root canal treatment failure is an inability to locate and adequately treat all canals of the root canal system [1]. In a statistical analysis of retreatment cases, Allen et al. [24] analyzed a total of 1300 endodontic subjects for factors that may have contributed to the failure of the original treatment and reported that untreated canals were responsible for failure in 114 cases, with 8.8% prevalence. In another investigation, Hoen and Pink [25] found that missed canals were the main cause of endodontic retreatment in 42% of cases studied. In our study, all evaluated teeth were two rooted. Unfortunately, the tooth bank at our dental school from which the examined teeth have been selected did not contain any three-rooted mandibular first molars. No studies have been conducted on the incidence of radix entomolaris and radix paramolaris in Palestine. Future in vivo studies are needed to investigate the prevalence of these anatomical variations.

Radiographic examination is an essential part of endodontic management, from initial diagnosis to monitoring treatment results. A periapical radiograph is the most common method used to assess the configuration of root canal systems during root canal treatments. It provides much needed information about root canal morphology especially if taken from different horizontal angels [26]. However, periapical radiographs produce a 2-dimensional image of 3-dimensional objects which prevents complete understanding of root morphology and canal anatomy [2].

Tooth clearing and canal staining had been considered for a long time the gold standard for studying root canal morphology where fine internal anatomic details can be visualized [27]. In this study, we used CBCT scanning to evaluate the number of canals and canal configuration of mandibular first molars. Neelakantan et al. [28] compared several methods used to evaluate root canal morphology and reported that CBCT was as accurate in identifying root canal systems as the modified canal staining and tooth clearing technique.

In fields of dentistry where 3-dimensional imaging is necessary, CBCT is considered by some to be the standard of care [29] with many advantages. CBCT is an office-based imaging technique so can be conveniently used in vivo when required for diagnosis and preoperative assessment. Additionally, CBCT scanning has been shown to be more accurate than digital radiographs in determining root canal systems. When compared with 2-dimensional digital radiographs, CBCT reduces or even eliminates the superimposition of the surrounding structures that normally overlap and enables clinicians to identify more canals in multicanal teeth that can then be instrumented and obturated, thereby increasing the likelihood of a successful outcome. Moreover, CBCT technology uses an extra-oral imaging scanner at a considerably lower radiation dose and higher resolution than conventional CT [18, 30].

The number of root canals and canal configuration of mandibular first permanent molars reported in the literature vary on the basis of the different ethnic populations and different methods used (Table 4). Our study showed that 54.4% of mandibular first molars had three canals and 41.3% had four. These results are in agreement with those of Zhang et al. [31] who reported an incidence of a fourth canal in 43% of a Chinese population. This value is lower than the findings of other previous studies. Chen et al. [17] reported an incidence of 45.9% in a Taiwanese population. In their study on mandibular molars in a Jordanian population using clearing technique, Al-Qudah and Awawdeh [12] reported a fourth canal in 46% of the studied sample. This incidence was 57.8% in a Saudi population [14].

The incidence of two canals in the mesial roots was found to be 95.6%. In their systematic review, de Pablo et al. [32] found a similar incidence (94.4%) in the 17 studies included accounting for 4535 mesial roots investigated.

In the present study, ten mesial roots (3.1%) were found to have three canals. The clinical significance of an extra canal means that failing to locate and properly treat this canal could lead to treatment failure. The presence of the middle mesial canal was reported up to 14.8% incidence [33]. In a study of 760 mandibular molars, Fabra-Campos [8] found that 20 (2.6%) teeth had three canals in the mesial root. In 13 of these (65%) the third canal joined the mesiobuccal canal in the apical third of the root and in 6 (30%) they converged with the mesiolingual canal, also in the apical third; the third canal ended as an independent canal in only 1 case. An overall same incidence (2.6%) was reported in the systematic review of de Pablo et al. [32].

In the mesial root, type IV configuration was most prevalent (53.8%) followed by type II (38.8%) canal configuration. These results are consistent with the findings of most of the earlier studies [5, 12, 15, 17]^.^ In their systematic review, de Pablo et al. [32] reported a prevalence in mesial roots of 52.3% for type IV and 35% with type II. An exception was reported by Al-Nazhan [14] with type II being the most prevalent followed by type IV. In the present study, ten mesial roots showed an additional configuration type (3-2) as described by Gulabivala et al. [27] and type (3-2-1) as described by Al-Qudah and Awawdeh [12]. Identification, preparation, and obturation of type IV and type II are relatively straightforward. However, the presence of additional types needs extra efforts, because failure to debride and disinfect this complex anatomy might have a direct effect on the treatment outcome.

The most prevalent canal configuration in the distal roots was type I (57.5%) followed by type II (22.5%), type III (10.6%), and type IV (8.1%). The high prevalence of the Vertucci type I canal configuration in the distal canals is consistent with the previous observations [12, 17, 27, 32]. In terms of type IV configuration, the incidence (8.1%) was lower than reported by previous studies [5, 12, 14, 17, 27].

In a Korean population, Kim et al. [34] reported a higher incidence (76.9%) of mesial roots with type IV and (66.6%) of distal roots with type I configuration. These higher incidences were also reported by Zhang et al. [31] in a Chinese population: 81% of mesial roots with type VI and 84% of distal roots with type I configuration. These differences may be related to study design (in vivo versus in vitro), study technique, sample size, and sample population.

The present study indicates that CBCT is helpful for the investigation of root canal morphology. When root canal assessment is possible from traditional periapical images or clinical procedures, the use of CBCT may not be necessary. When there are abnormal findings on traditional periapical films or variations detected clinically, it may be impossible to evaluate the root canal system effectively. In such situations, it is necessary to adopt CBCT for further diagnosis, whilst at the same time ensuring that the patients' exposure to radiation is kept as low as reasonably possible.

## 5. Conclusion

CBCT scans allow the identification of anatomic features and variations of the root canal system. Within the limitations of this study, it can be concluded that, in a Palestinian population, CBCT scans show that mandibular first permanent molars commonly have four canals with a lower incidence (41.3%) compared to previously reported data. Therefore, to treat mandibular first permanent molars, dentists need to be aware of the possible existence of a separate distolingual root canal before they initiate endodontic treatment.

## Figures and Tables

**Table 1:** Number and type of canals per examined teeth.

Number of canals	Number of teeth (%)
2 (1 mesial, 1 distal)	4 (1.2)
3 (2 mesials, 1 distal)	174 (54.4)
4 (2 mesials, 2 distals)	132 (41.3)
4 (3 mesials, 1 distal)	4 (1.2)
5 (3 mesials, 2 ditals)	6 (1.9)

Total	320 (100)

**Table 2:** Number and percentage of canals per examined root.

Number of canals	Mesial root (%)	Distal root (%)
1	4 (1.3)	184 (57.5)
2	306 (95.6)	136 (42.5)
3	10 (3.1)	0

Total	320 (100)	320 (100)

**Table 3:** Types of canal configuration per root.

Number of canals	Canal configuration					
	type I	II	III	IV	V	additional type
Root						
Mesial	4	124	6	172	4	10∗
Total = 320	1.15%	38.8%	1.9%	53.8%	1.15%	3.2%
Distal	184	72	34	26	4	0
Total = 320	57.5%	22.5%	10.6%	8.1%	1.3%	

^*^8 roots has type (3-2) configuration (type 12 according to Gulabivala et al. [27] classification) and 2 roots has type (3-2-1) configuration (type 22 according to Al-Qudah and Awawdeh [12] classification).

**Table 4:** Summary of some previous studies of the canal number and canal configuration.

Author (population)	No. of teeth	No. of canals (%)		Mesial root canal configuration %		Distal root canal configuration %		
		3	4	type II	type IV	type I	type II	type III
Al-Qudah and Awawdeh [12] (Jordan)	330	48	46	36	53	54	17	9.4
Al-Nazhan [14] (Saudi)	251	42	58	52.6	47.4	42.2	35	22.7
Chen et al. [17] (Taiwanese)	183	51	46	29.5	55.2	54	12.6	25
Gulabivala et al. [27] (Thai)	118	61	30.5	21	51	61	4.2	15.3
Zhang et al. [31] (Chinese)	232	56	43	—	81	84	—	—
Kim et al. [34] (Korean)	976	48	49	19	77	66	19	12
